# Prognostic nutritional index predicts 1-year hospitalization and mortality in outpatients with cirrhosis

**DOI:** 10.17305/bb.2026.14035

**Published:** 2026-04-02

**Authors:** Ayşe Karaduru Avcı, Zeynep Gök Sargın, Fatih Sargın

**Affiliations:** 1 Department of Internal Medicine, The Antalya City Hospital, Antalya, Türkiye; 2 Department of Gastroenterology, The Antalya City Hospital, Antalya, Türkiye; 3 Department of Medical Intensive Care Unit, The Antalya City Hospital, Antalya, Türkiye

**Keywords:** Cirrhosis, prognostic nutritional index, malnutrition, hospitalization, prognosis

## Abstract

Malnutrition is prevalent in patients with cirrhosis and is linked to negative clinical outcomes. However, research on the prognostic nutritional index (PNI) and the burden of hospitalization over one year in stable outpatients remains limited. This study aimed to assess the relationship between PNI and liver-related hospitalization outcomes, as well as 12-month mortality, in outpatients with cirrhosis. In this retrospective single-center study, 125 adult patients with stable cirrhosis were analyzed, focusing on baseline demographic, clinical, laboratory, and 12-month follow-up data. Logistic regression was employed to evaluate the incidence of hospitalization in the entire cohort, while Poisson regression assessed hospital admission counts among patients with complete follow-up, and linear regression investigated the total length of stay for hospitalized patients. The results indicated that lower PNI values were significantly correlated with an increased likelihood of hospitalization and a higher frequency of hospital admissions within one year. In multivariable analysis, PNI remained an independent predictor of hospitalization (odds ratio [OR] ═ 0.655, 95% confidence interval [CI]: 0.530–0.809, *P <* 0.001) and admission frequency (incidence rate ratio [IRR] ═ 0.90, 95% CI: 0.848–0.955, *P <* 0.001), while no significant association was found with the length of hospital stay. Each 5-unit increase in PNI corresponded to substantially lower odds of hospitalization (OR = 0.121, 95% CI: 0.042–0.347) and a reduced admission rate (IRR = 0.59, 95% CI: 0.444–0.794). Additionally, PNI exhibited significant negative correlations with the Model for End-Stage Liver Disease–Sodium (MELD-Na) and Child–Turcotte–Pugh scores (all *P <* 0.001). PNI also demonstrated strong predictive validity for 12-month cirrhosis-related mortality, with an area under the curve (AUC) of 0.873 (95% CI: 0.802–0.943) and an optimal cut-off value of 38.5. Patients with a PNI ≤38.5 experienced significantly reduced 12-month survival (log-rank *P <* 0.001). These findings suggest that PNI may serve as a straightforward and accessible marker for nutritional assessment and early risk stratification in stable outpatients with cirrhosis.

## Introduction

Liver diseases account for approximately 4% of all deaths globally, with cirrhosis being a predominant cause of liver-related mortality [1]. The clinical progression of cirrhosis can vary, ranging from an asymptomatic compensated stage to a decompensated stage [2]. Most mortality and morbidity occur during the decompensated phase, characterized by increased hospital admissions, medical interventions, and medication usage. In advanced stages, the requirement for liver transplantation often arises, imposing a significant burden on patient management and the healthcare system [3].

Malnutrition is prevalent among patients with cirrhosis and heightens the risk of complications such as liver failure, infections, hepatic encephalopathy (HE), and ascites. Furthermore, it adversely affects the survival of patients undergoing liver transplantation [4]. The Prognostic Nutritional Index (PNI), which reflects nutritional status [5], serves as a simple and applicable biomarker associated with the development of complications and mortality in cirrhotic patients [6].

To our knowledge, limited data are available regarding the relationship between PNI and the one-year hospitalization burden in stable cirrhotic outpatients. This study aims to expand the existing evidence by focusing specifically on stable outpatient cirrhosis and its associated hospitalization burden over one year.

## Materials and methods

### Study design and ethical approval

This retrospective observational study was conducted at Antalya City Hospital from December 2023 to September 2024. We screened patients aged ≥18 years with a diagnosis of liver cirrhosis who were followed in the gastroenterology outpatient clinic for eligibility.

The study protocol received approval from the Antalya City Hospital Ethics Committee (decision number 11/76, dated 28.08.2025). The research adhered to the principles of the Declaration of Helsinki and relevant regulations.

### Study population

Eligible participants included patients aged ≥18 years diagnosed with liver cirrhosis, who were under follow-up in the gastroenterology outpatient clinic and had available baseline clinical and laboratory data, including PNI. The diagnosis of cirrhosis was based on a combination of clinical findings, laboratory parameters, imaging studies (ultrasound and/or elastography), and, when available, histopathological confirmation.

Exclusion criteria included patients with active infections, a history of malignancy, prior liver transplantation, missing or inaccessible data, and those lost to follow-up. Active infection was defined by the presence of at least one of the following: fever (>38^∘^C), leukocytosis (>10,000/mm^3^), elevated C-reactive protein levels (>10 mg/L), or clinical/microbiological evidence of infection. Patients lost to follow-up were those who did not attend any follow-up visits during the 12-month observation period or for whom sufficient data could not be obtained from electronic medical records.

This study specifically focused on stable cirrhosis patients, defined as individuals without active decompensation at the time of the outpatient visit. Stable cirrhosis was operationally defined as the absence of active variceal bleeding, overt HE (West Haven grade ≥2), clinically significant ascites (requiring medical treatment or therapeutic paracentesis), spontaneous bacterial peritonitis (SBP), or hepatorenal syndrome (HRS) at baseline. Notably, patients with a history of prior decompensation were not excluded.

The overall cohort included both survivors and non-survivors (*n* ═ 125). Hospitalization within one year was evaluated in the entire cohort, including patients who died during follow-up. However, hospitalization-related outcomes requiring complete follow-up (e.g., total length of stay and number of admissions) were analyzed only in patients who completed the 12-month follow-up period (*n* ═ 107) to ensure comparable observation time and minimize bias related to unequal follow-up duration.

### Data collection and clinical evaluation

Baseline clinical and laboratory data were collected during the index outpatient visit. Nutritional status was assessed using the PNI, calculated as follows:

PNI = 10 × albumin (g/dL) + 0.005× total lymphocyte count (/mm^3^) [5].

The 12-month follow-up period was defined as the time interval beginning with the index visit, during which baseline data and PNI were obtained, and extending up to 12 months thereafter. Clinical records during this period were retrospectively reviewed using the hospital information management system.

Demographic characteristics (age, sex), cirrhosis etiology, comorbidities, and laboratory parameters were recorded.

Hospitalization outcomes included only liver-related admissions due to cirrhosis-related complications, such as ascites, SBP, HE, gastrointestinal variceal bleeding, and HRS. Non-liver-related admissions (e.g., cardiovascular events, elective procedures, unrelated infections) were excluded. Hospitalizations were identified through electronic medical records, discharge summaries, and the national health database system (e-Nabız), and were reviewed by the study investigators.

Cirrhosis-related complications were recorded as binary variables, indicating the presence or absence of at least one episode during the 12-month follow-up period. Given the relatively low number of events, complication outcomes were considered exploratory and not defined as primary study endpoints.

Mortality was defined as cirrhosis-related mortality occurring within the 12-month follow-up period, as determined from clinical records, discharge summaries, and documented causes of death in the hospital information system.

Disease severity was assessed using the Child–Turcotte–Pugh (CTP) [7] and Model for End-Stage Liver Disease Sodium (MELD-Na) [8] scores.

### Statistical analysis

Statistical analyses were performed using R software (version 4.3.1, 2023-06-16, ucrt). Categorical data are summarized as numbers (percentages) (n (%)). Continuous variables are presented as mean ± standard deviation and median (interquartile range, IQR), depending on their distribution. Normal distribution was assessed using kurtosis-skewness coefficients, the Kolmogorov–Smirnov test, and the Shapiro–Wilk test. Group comparisons for non-normally distributed variables were conducted using the Mann–Whitney *U* test. Correlations between numerical variables were evaluated using Spearman’s correlation analysis.

To clarify the analysis populations, different analysis sets were defined *a priori*: (1) the entire cohort (*n* ═ 125) for hospitalization presence and mortality analyses; (2) patients with complete 12-month follow-up (*n* ═ 107) for admission count analyses; and (3) hospitalized patients (*n* ═ 48) for length-of-stay analyses.

Patients who died during follow-up were included in analyses of hospitalization presence but excluded from analyses requiring complete follow-up (admission counts and length of stay) to ensure comparable observation time across individuals.

The presence of hospitalization within one year was analyzed using logistic regression for the entire cohort (*n* ═ 125). Logistic regression analysis identified factors associated with hospitalization presence, with results expressed as odds ratios (ORs) and 95% confidence intervals.

Linear regression analysis evaluated factors associated with total length of hospital stay among hospitalized patients. Regression coefficients (β) with 95% confidence intervals were reported (*n* ═ 48). Multivariable linear regression was not performed due to only one significant variable identified in univariable analysis.

The number of hospital admissions was analyzed using univariable and multivariable Poisson regression models in patients who completed 12-month follow-up (*n* ═ 107). Poisson regression assessed the association between clinical variables and the number of hospital admissions within one year, with results expressed as incidence rate ratios (IRRs) and 95% confidence intervals. Model fit was evaluated using goodness-of-fit statistics, including Pearson chi-square and deviance divided by their degrees of freedom. No evidence of overdispersion was observed (Pearson χ^2^/df = 0.62; deviance/df = 0.35), supporting the use of the Poisson model.

Variables included in multivariable models were selected based on clinical relevance and supported by univariable analysis findings.

Survival analysis utilized the Kaplan–Meier method, with differences between groups assessed using the log-rank test according to PNI categories. All survival analyses were focused on 12-month cirrhosis-related mortality. Receiver operating characteristic (ROC) analysis was conducted to evaluate the discriminative ability of PNI for 12-month cirrhosis-related mortality in the entire cohort (*n* ═ 125).

Multicollinearity among variables in the multivariable model was assessed using variance inflation factor (VIF) values, with a VIF value >5 indicating significant multicollinearity.

For enhanced clinical interpretability, effect estimates for PNI were additionally expressed per 5-unit increase using the exponential transformation of regression coefficients.

Statistical significance was set at *P <* 0.05 for all tests. Observations with missing data were excluded from the analyses. Due to the retrospective design and limited patient sample, an *a priori* power analysis was not feasible. The sample size was determined based on the number of eligible patients during the study period, necessitating cautious interpretation of results in light of potential type II error risks.

## Results

### Baseline characteristics

The study included 125 patients diagnosed with liver cirrhosis, all of whom were monitored for a minimum of one year. The mean age of the participants was 61.66 ± 12.98 years, with 64% (*n* ═ 80) being male. The predominant etiology was cryptogenic cirrhosis (36%), followed by hepatitis B (26.4%) and alcohol-related cirrhosis (18.4%). By the conclusion of the follow-up period, 18 patients (14.4%) had died. The demographic, clinical, and laboratory characteristics of the patients are detailed in Table 1.

**Table 1:** Baseline characteristics and one-year follow-up outcomes of patients with liver cirrhosis (*n* ═ 125)

**Variable**	***n* (%) / Mean ± SD**	**Median (IQR)**
Demographic characteristics		
Age (years)	61.66 ± 12.98	62 (53–71)
Sex		
Male	80 (64.0)	–
Etiology of cirrhosis, *n* (%)		
Cryptogenic	45 (36.0)	–
Alcohol-related	23 (18.4)	–
HBV	33 (26.4)	–
HCV	1 (0.8)	–
Autoimmune hepatitis	6 (4.8)	–
Congestive heart failure-related	5 (4.0)	–
NASH	12 (9.6)	–
Comorbidities, *n* (%)		
Diabetes mellitus	42 (33.6)	
Hypertension	44 (35.2)	
Laboratory parameters		
Hemoglobin (g/dL)	11.57 ± 2.80	11 (9.5–14.0)
Platelet count (×10^3^/µL)	137.6 ± 73.7	125.0 (90.0–170.0)
Lymphocyte count (×10^3^/µL)	1.46 ± 0.67	1.40 (0.96–1.8)
Neutrophil count (×10^3^/µL)	4.25 ± 3.6	3.40 (2.50–4.90)
Serum albumin (g/dL)	3.42 ± 0.77	3.5 (2.8–4.0)
Creatinine (mg/dL)	0.94 ± 0.31	0.9 (0.75–1.0)
Estimated GFR (mL/min/1.73m^2^)	78.76 ± 20.99	82 (66–91)
Disease severity scores		
Prognostic nutritional index	41.53 ± 9.58	42 (34.5–48.5)
MELD-Na score	14.39 ± 5.55	13 (10–17)
CTP score	6.81 ± 2.25	6 (5–8)
One-year outcomes		
Mortality, *n* (%)	18 (14.4)	
Hospitalization ≥1, *n* (%)	48 (38.4)	
Total length of hospital stay, days (1 year)	3.45 ± 6.89	0 (0–5)
Ascites ≥1 episode, *n* (%)	24 (19.2)	
HE ≥1 episode, *n* (%)	15 (12.0)	
SBP ≥1 episode, *n* (%)	2 (1.6)	
Variceal bleeding ≥1, *n* (%)	17 (13.6)	
HRS ≥1 episode, *n* (%)	4 (3.2)	

Categorical variables are reported as *n* (%), while continuous variables are presented as mean ± standard deviation or median (interquartile range), depending on the context. **Abbreviations:** CTP, Child–Turcotte–Pugh; GFR, glomerular filtration rate; HBV, hepatitis B virus; HCV, hepatitis C virus; HE, hepatic encephalopathy; HRS, hepatorenal syndrome; IQR, interquartile range; MELD-Na, Model for End-Stage Liver Disease Sodium; NASH, nonalcoholic steatohepatitis; PNI, prognostic nutritional index; SBP, spontaneous bacterial peritonitis; SD, standard deviation.

### Comparison between survivors and non-survivors

In comparing mortality groups, the median age (IQR) was significantly higher among non-survivors than survivors (72 [63]–[77] vs. 61 [51.5–69.5] years, *P ═* 0.008). Hemoglobin, lymphocyte count, and albumin levels were lower in non-survivors, while MELD-Na and CTP scores, along with hospitalization frequency and duration, were higher. The mean PNI was significantly lower in non-survivors compared to survivors (31.22 ± 5.1 vs. 43.27 ± 9.06, *P <* 0.001). Detailed comparisons are presented in Table 2.

**Table 2:** Comparative analysis of demographic, laboratory, and clinical parameters between survivors and non-survivors (*n* ═ 125)

**Variable**	**Survivors (*n* ═ 107) Mean ± SD**	**Median (IQR)**	**Non-survivors (n = 18) Mean ± SD**	**Median (IQR)**	***P* value**
Age (years)	60.45 ± 12.68	61 (51.5–69.5)	68.83 ± 12.74	72 (63–77)	0.008
Hemoglobin (g/dL)	11.86 ± 2.78	12 (10–14)	9.90 ± 2.31	9.15 (8.77–10.2)	0.003
Platelet count (×10^3^/µL)	139.6 ± 77.0	125.0 (90.0–162.0)	125.8 ± 50.3	130.0 (76.0–178.3)	0.814
Lymphocyte count (×10^3^/µL)	1.56 ± 0.67	1.5 (1.1–2.0)	0.88 ± 0.28	0.8 (0.7–1.0)	<0.001
Neutrophil count (×10^3^/µL)	4.11 ± 3.73	3.3 (2.5–4.2)	5.09 ± 2.61	4.5 (3.2–6.0)	0.015
Serum albumin (g/dL)	3.55 ± 0.74	3.7 (3.0–4.1)	2.68 ± 0.48	2.65 (2.5–2.88)	<0.001
Creatinine (mg/dL)	0.91 ± 0.26	0.88 (0.74–1.0)	1.14 ± 0.47	1.0 (0.9–1.15)	0.014
GFR (mL/min/1.73m^2^)	80.93 ± 20.0	83 (69–94)	65.83 ± 22.64	66.5 (50.75–85.75)	0.016
MELD-Na score	13.38 ± 4.91	12 (10–15)	20.39 ± 5.46	20.5 (18–24.75)	<0.001
CTP score	6.34 ± 2.00	5 (5–7)	9.61 ± 1.50	10 (9–10)	<0.001
PNI	**43.27 ± 9.06**	44 (36.5–49.5)	**31.22 ± 5.18**	30.75 (28.62–34.12)	<0.001
Clinical outcomes					
Hospitalization ≥1, *n* (%)	30 (28.0)		18 (100.0)		<0.001
Hospitalization frequency (1 year), *n* (%)					<0.001
0 admissions 1 admission ≥2 admissions	77 (72.0) 21 (19.6) 9 (8.4)		0 (0.0) 8 (44.4) 10 (55.6)		
Hospital stay (days, 1 year)	1.67 ± 3.17	0 (0–2.5)	14.0 ± 12.09	11 (4.25–19.25)	<0.001
Ascites ≥1 episode, *n* (%)	11 (10.3)		13 (72.2)		<0.001
HE ≥1 episode, *n* (%)	7 (6.5)		8 (44.4)		<0.001
SBP ≥1 episode, *n* (%)	2 (1.9)		0 (0.0)		0.732
Variceal bleeding ≥1, *n* (%)	13 (12.1)		4 (22.2)		0.209
HRS ≥1 episode, *n* (%)	0 (0.0)		4 (22.2)		<0.001

Categorical variables are presented as *n* (%), while continuous variables are expressed as mean ± standard deviation or median (interquartile range), depending on appropriateness. **Abbreviations:** CTP, Child–Turcotte–Pugh; GFR, glomerular filtration rate; HE, hepatic encephalopathy; HRS, hepatorenal syndrome; IQR, interquartile range; MELD-Na, Model for End-Stage Liver Disease Sodium; PNI, prognostic nutritional index; SBP, spontaneous bacterial peritonitis; SD, standard deviation.

### Correlation analysis

PNI was negatively correlated with hospitalization frequency (r ═ –0.76), MELD-Na (r ═ –0.64), and CTP score (r ═ –0.82) (all *P <* 0.001) (Table 3).

**Table 3:** Correlation between PNI and clinical/laboratory parameters (Spearman test) (*n* ═ 125)

**Variable**	**Correlation coefficient (r)**	***P* value**
Age	−0.005	0.96
1-year hospitalizations	−0.761	**< 0.001**
Hospital stay (days)	−0.77	**< 0.001**
MELD-Na	−0.639	**< 0.001**
CTP	−0.816	**< 0.001**

**Abbreviations:** CTP, Child–Turcotte–Pugh; MELD-Na, Model for End-Stage Liver Disease Sodium; PNI, prognostic nutritional index.

### Predictors of hospitalization

The primary outcome of this study was hospitalization within one year. In the entire cohort (*n* ═ 125), including patients who died during follow-up, logistic regression analysis demonstrated that lower PNI and hemoglobin levels were independently associated with the likelihood of hospitalization. In the multivariable model, the PNI emerged as an independent predictor of hospitalization, with an OR of 0.655 and a 95% confidence interval (CI) ranging from 0.530–0.809 (*P <* 0.001) (Table 4). The OR for PNI (OR = 0.655) indicates that each 1-unit increase in PNI is associated with a decreased likelihood of hospitalization.

**Table 4:** Logistic regression analysis of hospitalization within one year (*n* ═ 125)

**Variable**	**Univariable OR**	**95% CI (Univariable)**	***P***	**Multivariable OR**	**95% CI (Multivariable)**	***P***
Age (years)	0.999	0.971–1.027	0.926	1.024	0.954–1.099	0.512
Hemoglobin (g/dL)	0.471	0.360–0.616	<0.001	0.461	0.288–0.739	0.001
Platelet per 10 ×10^3^/µL increase	0.996	0.991–1.002	0.171	–	–	–
Lymphocyte (×10^3^/µL)	0.998	0.997–0.999	<0.001	–	–	–
Albumin (g/dL)	0.018	0.004–0.069	<0.001	–	–	–
PNI	0.635	0.536–0.753	<0.001	0.655	0.530–0.809	<0.001
GFR	0.990	0.973–1.007	0.250	–	–	–
MELD-Na score	1.374	1.223–1.543	<0.001	1.181	0.982–1.420	0.077
CTP score	3.314	2.249–4.884	<0.001	–	–	–

**Abbreviations:** CI, confidence interval; CTP, Child–Turcotte–Pugh; GFR, glomerular filtration rate; MELD-Na, Model for End-Stage Liver Disease Sodium; OR, odds ratio; PNI, prognostic nutritional index.

When expressed as a 5-unit increase in PNI, the odds of hospitalization were significantly reduced (OR = 0.121, 95% CI: 0.042–0.347), reflecting an approximate 88% reduction in hospitalization rates.

No evidence of multicollinearity was detected among variables in the multivariable model (all VIF values < 2.1). Specifically, VIF values were 1.10 for age, 1.59 for hemoglobin, 2.10 for PNI, and 1.73 for MELD-Na.

### Length of hospital stay

Among patients with at least one hospitalization (*n* ═ 48), univariable linear regression analysis indicated that age was significantly associated with total length of hospital stay (β ═ 0.193, 95% CI: 0.037–0.350, *P ═* 0.016), whereas PNI did not show a significant association with length of stay (Table 5).

**Table 5:** Univariable linear regression analysis for total length of hospital stay among hospitalized patients (*n* ═ 48)

**Variable**	**β**	**95% CI**	*P*
Age (years)	0.193	0.037–0.350	0.016
Hemoglobin (g/dL)	−0.157	−1.332–1.018	0.789
Platelet per 10 ×10^3^/µL increase	0.001	−0.032–0.033	0.954
Lymphocyte (×10^3^/µL)	−0.003	−0.008–0.003	0.351
Albumin (g/dL)	−0.453	−5.641–4.734	0.861
PNI	−0.133	−0.617–0.350	0.581
GFR	−0.087	−0.194–0.020	0.109
MELD-Na	−0.002	−0.460–0.456	0.994
CTP	0.029	−1.316–1.374	0.966

Values are presented as unstandardized β coefficients with corresponding 95% CIs. The analysis was conducted exclusively among patients who experienced at least one hospitalization. **Abbreviations:** CI, confidence interval; CTP, Child–Turcotte–Pugh; GFR, glomerular filtration rate; MELD-Na, Model for End-Stage Liver Disease Sodium; PNI, prognostic nutritional index.

Multivariable analysis was not conducted due to limited statistical power.

### Hospital admission counts (poisson analysis)

In patients with complete follow-up (*n* ═ 107), excluding those who died during follow-up, Poisson regression analysis revealed that lower PNI and hemoglobin levels were independently associated with a higher number of hospital admissions within one year. In the multivariable model, PNI (IRR = 0.90, 95% CI: 0.848–0.955, *P <* 0.001) and hemoglobin (IRR = 0.815, 95% CI: 0.697–0.953, *P ═* 0.010) remained significant predictors, while age and MELD-Na were not significantly associated with admission counts (Table 6).

**Table 6:** Poisson regression analysis of one-year hospital admission counts (*n* ═ 107)

**Variable**	**Univariable** **IRR**	**95% CI (Univariable)**	***P***	**Multivariable** **IRR**	**95% CI (Multivariable)**	***P***
Age (years)	0.981	0.957–1.006	0.130	1.007	0.981–1.034	0.596
Hgb (g/dL)	0.682	0.607–0.766	**<0.001**	0.815	0.697–0.953	**0.010**
Platelet per 10 ×10^3^/µL increase	0.995	0990–1.001	0.085			
Lym (×10^3^/µL)	0.999	0.998–0.999	**<0.001**			
Albumin (g/dL)	0.246	0.164–0.369	**<0.001**			
PNI	0.880	0850–0.912	**<0.001**	0.900	0.848–0.955	**<0.001**
GFR	1.005	0.989–1.021	0.565			
MELD-Na score	1.125	1.074–1.178	**<0.001**	0.982	0.907–1.062	0.645
CTP score	1.431	1.287–1.591	**<0.001**			

**Abbreviations:** CI, confidence interval; CTP, Child–Turcotte–Pugh; GFR, glomerular filtration rate; Hgb, hemoglobin; IRR, incidence rate ratio; Lym, lymphocyte count; MELD-Na, Model for End-Stage Liver Disease Sodium; PNI, prognostic nutritional index.

When expressed as a 5-unit increase in PNI, this corresponded to a lower rate of hospital admissions (IRR = 0.59, 95% CI: 0.444–0.794), indicating an approximate 41% reduction in admission rates.

### ROC analysis

In a cohort of 125 participants, ROC analysis revealed that the PNI has strong discriminatory power in predicting 12-month cirrhosis-related mortality, with an area under the curve (AUC) of 0.873 (95% CI: 0.802–0.943). The optimal cut-off value was identified as 38.5, with a sensitivity of 94.4% (95% CI: 74.24–99.01), specificity of 70.1% (95% CI: 60.84–77.95), positive predictive value of 34.7%, and negative predictive value of 98.7% (Figure 1).

**Figure 1.**
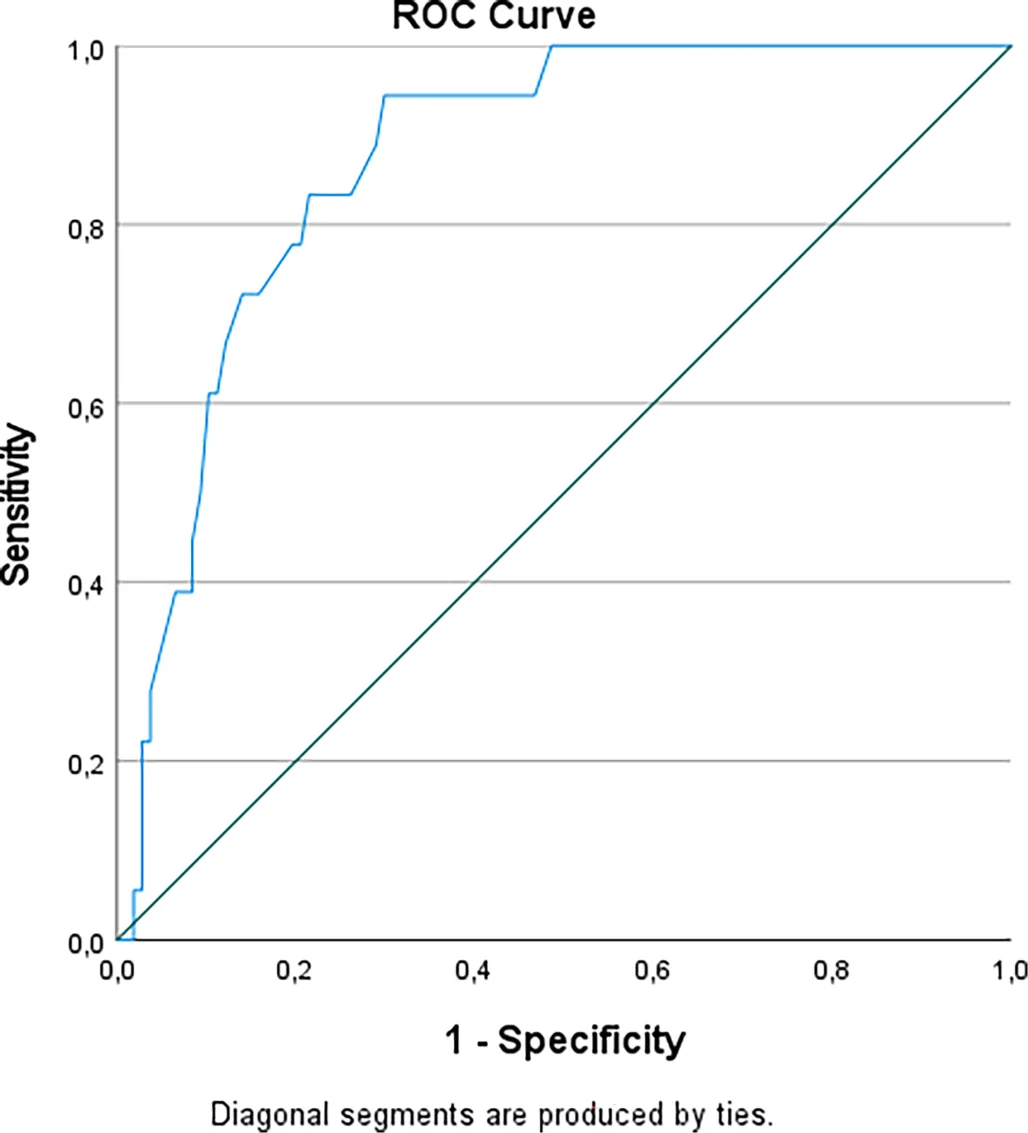
**Receiver operating characteristic curve of the PNI for predicting 12-month cirrhosis-related mortality in the overall cohort (*n* ═ 125).** The Prognostic Nutritional Index showed good discriminative ability for 12-month cirrhosis-related mortality, with an AUC of 0.873 (95% CI: 0.802–0.943). The optimal cut-off value was 38.5, yielding a sensitivity of 94.4%, specificity of 70.1%, positive predictive value of 34.7%, and negative predictive value of 98.7%. The diagonal line represents the line of no discrimination. **Abbreviations:** AUC, area under the curve; CI, confidence interval; PNI, Prognostic Nutritional Index; ROC, receiver operating characteristic.

### PNI cut-off–based comparisons

Patients with PNI ≤ 38.5 exhibited higher rates of mortality, hospitalization, and complications compared to those with PNI > 38.5. Specifically, mortality rates were 34.7% in the low-PNI group vs 1.3% in the high-PNI group, while hospitalization rates were 87.8% vs 6.6%, respectively. Additionally, complications such as ascites (42.9% vs. 3.9%) and variceal bleeding (34.7% vs. 0%) were more frequent in the low-PNI group. These findings should be interpreted with caution due to the relatively low number of events for some complication outcomes. Detailed comparisons are presented in Table 7.

**Table 7:** Clinical outcomes by PNI groups (Cut-off: 38.5) (*n* ═ 125)

**Variable**	**PNI ≤ 38.5 (*n* ═ 49)**	**PNI > 38.5 (*n* ═ 76)**	***P* value**
Mortality, *n* (%)	17 (34.7)	1 (1.3)	<0.001
Hospitalization ≥1, *n* (%)	43 (87.8)	5 (6.6)	<0.001
Ascites ≥1 episode, *n* (%)	21 (42.9)	3 (3.9)	<0.001
HE ≥1 episode, *n* (%)	12 (24.5)	3 (3.9)	0.002
SBP ≥1 episode, *n* (%)	2 (4.1)	0 (0.0)	0.152
Variceal bleeding ≥1, *n* (%)	17 (34.7)	0 (0.0)	<0.001
HRS ≥1 episode, *n* (%)	4 (8.2)	0 (0.0)	0.022

Categorical variables are reported as n (%), while complications are defined as the occurrence of at least one episode during the 12-month follow-up period. **Abbreviations:** HE, hepatic encephalopathy; HRS, hepatorenal syndrome; PNI, prognostic nutritional index; SBP, spontaneous bacterial peritonitis.

### Survival analysis

Patients with PNI ≤ 38.5 demonstrated significantly lower 12-month survival compared to those with PNI >38.5 (log-rank χ^2^ ═ 28.45, *P <* 0.001), corroborating the observed differences in 12-month cirrhosis-related mortality between groups. Mortality was markedly higher in the low-PNI group (17/49 [34.7%]) compared to the high-PNI group (1/76 [1.3%]) (Figure 2).

**Figure 2.**
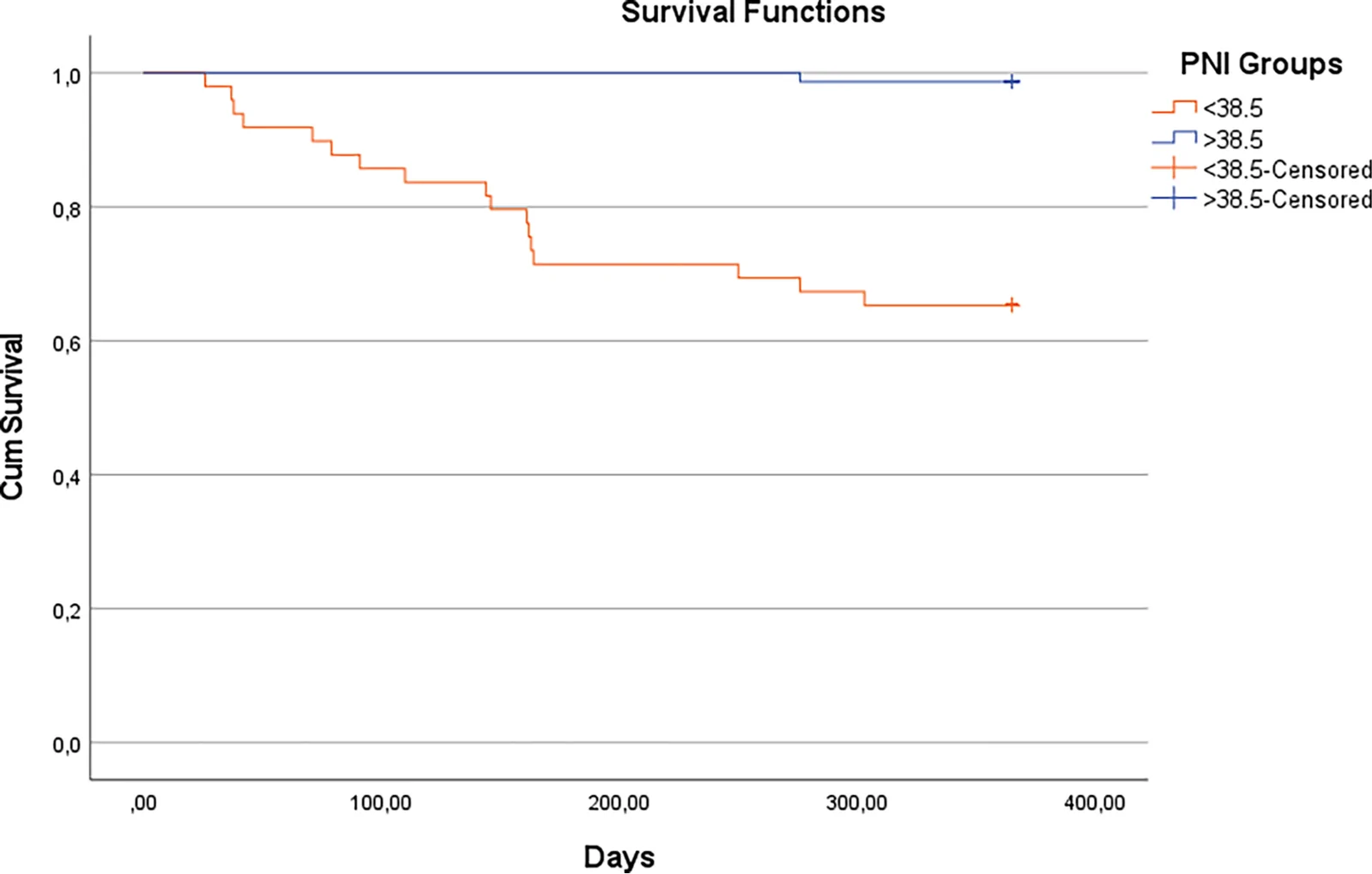
**Kaplan–Meier survival curves for 12-month cirrhosis-related survival according to PNI groups (cut-off: 38.5) in the overall cohort (*n* ═ 125).** Patients with PNI ≤38.5 had significantly lower cumulative 12-month survival than those with PNI >38.5 (log-rank χ^2^ ═ 28.45, *P <* 0.001). Mortality was markedly higher in the low-PNI group than in the high-PNI group (17/49 [34.7%] vs. 1/76 [1.3%]). Tick marks indicate censored observations. **Abbreviation:** PNI, prognostic nutritional index.

## Discussion

This study utilized PNI to evaluate the nutritional status of patients with cirrhosis. Our findings indicate that lower PNI values correlate with poorer clinical outcomes. Specifically, lower PNI levels were independently linked to an increased likelihood of hospitalization and a higher number of hospital admissions within one year. In multivariable analysis, PNI remained a significant predictor of hospitalization. Furthermore, PNI was associated with admission frequency, suggesting that poor nutritional status may be related to both the occurrence and the burden of hospitalizations. However, no significant association was found between PNI and length of hospital stay among hospitalized patients.

Kaplan–Meier analysis illustrated a clear differentiation in survival curves, with higher mortality observed in patients with PNI ≤ 38.5. These findings support the discriminative ability of PNI in this cohort. Additionally, lower PNI values correlated with higher MELD-Na and CTP scores. The negative correlations between PNI and these severity indices, along with the absence of significant multicollinearity, suggest that PNI may reflect overall disease burden and could provide supplementary information alongside established severity scores.

Malnutrition is prevalent among patients with cirrhosis and heightens the risk of complications, with prevalence rates ranging from 20% to 50% [4]. While malnutrition may often go unnoticed in patients with compensated cirrhosis due to subtle symptoms, nutritional deficiencies in the decompensated stage are associated with increased risk of complications and mortality [9]. This situation leads to heightened healthcare utilization and negatively impacts quality of life. The causes of malnutrition are multifaceted, with reduced oral intake, loss of appetite, cognitive impairment, and frequent hospital admissions among the primary contributing factors [10, 11]. Micronutrient deficiencies may accompany cirrhosis, particularly in alcohol-related liver disease [12]. In cholestatic liver diseases, deficiencies of fat-soluble vitamins may arise due to impaired bile flow [13]. Notably, deficiencies in vitamin D and zinc are clinically significant; vitamin D deficiency is linked to muscle loss and frailty, while zinc deficiency is associated with HE and sarcopenia [14, 15]. Therefore, early identification of malnutrition, appropriate vitamin-mineral supplementation, and nutrition interventions guided by a dietitian may play a crucial role in mitigating complications and enhancing survival.

Recent advancements in medical technology, particularly the integration of the Internet of Things (IoT), have created new avenues for remote monitoring. In patients with cirrhosis, these approaches may facilitate early detection of clinical deterioration and support timely interventions, potentially improving follow-up care and reducing hospitalization rates [16].

The PNI is a straightforward parameter derived from albumin and total lymphocyte counts, reflecting both malnutrition and inflammatory processes [5]. In patients with cirrhosis, low albumin levels signify impaired liver function and systemic inflammation, while decreased lymphocyte counts indicate immune deficiency [17, 18]. Consequently, the PNI may offer complementary insights beyond albumin or lymphocyte values alone. The literature highlights that a poor prognosis in cirrhosis cannot be solely attributed to hepatic insufficiency; inflammation also plays a crucial role in determining the severity of complications and survival [19]. In this context, low PNI values may indicate poor prognosis as a combined marker of malnutrition and heightened inflammatory burden.

Previous studies have established the prognostic significance of the PNI across various conditions, including liver diseases, cancer, cardiovascular diseases, and metabolic disorders [20–23]. In 2023, Xie et al. assessed the 3- and 6-month mortality predictions associated with the PNI in patients with decompensated cirrhosis, revealing that a low PNI correlated with reduced survival and demonstrated a significant negative correlation with the MELD-Na score [6]. Consistent with these findings, our study reported a strong negative correlation of the PNI with MELD-Na and CTP scores (r ═ –0.64 and r ═ –0.82, *P <* 0.001) in a more stable cirrhotic population during outpatient follow-up. However, the absence of an independent effect of MELD-Na in the multivariable model warrants cautious interpretation, given the sample size and study design.

Ao et al. conducted a retrospective observational study encompassing a broad patient cohort, including both compensated and decompensated cirrhosis cases, to evaluate the prognostic value of the PNI [24]. They found that the PNI was significantly associated not only with the severity of cirrhosis but also with the development of complications, with particularly low PNI levels correlating with a poor prognosis. Additionally, they identified platelet count, alongside PNI, as an independent marker of poor prognosis [24]. In contrast, our study found no significant differences in platelet counts between mortality groups, and platelet count did not exhibit a statistically significant effect on one-year hospitalization parameters. This discrepancy may be attributed to the smaller cohort size, the stable patient group, and the heterogeneity of cirrhosis etiologies in our study.

A recent prospective study by Santos et al. introduced a nutritional prognostic index (NPI), derived from parameters similar to those of the PNI, which was linked to mortality in patients with advanced chronic liver disease [25]. The authors underscored the clinical relevance of nutrition-based indices in risk assessment. In our investigation of a stable outpatient cirrhosis population, the PNI exhibited strong discriminative ability for predicting mortality. Furthermore, lower PNI levels were independently associated with increased likelihood of hospitalization and frequency of hospital admissions. The optimal threshold identified in our study (38.5) differed from that reported by Santos et al., potentially due to variations in patient population, disease severity, and nutritional status.

In conclusion, our study indicates that the PNI may serve as a valuable marker for assessing nutritional status and for risk stratification in patients with stable cirrhosis. Lower PNI values were consistently associated with a higher risk of hospitalization, increased healthcare utilization, and poorer survival outcomes. However, these findings should be interpreted as associations observed in a retrospective, single-center study. Additionally, findings related to complications should be considered exploratory due to the relatively low number of events.

While prior studies have predominantly focused on hospitalized or decompensated cirrhosis populations, our findings contribute to the literature by evaluating the PNI in a stable outpatient cohort and by addressing both hospitalization burden and survival outcomes. These results suggest that the PNI may act as a complementary marker in the clinical assessment of patients with cirrhosis.

Despite the promising results, our study has limitations. First, the small sample size may limit the generalizability of our findings. Second, as the study was conducted at a single center, the results may not apply to other healthcare settings, geographic regions, or patient populations with diverse demographic and etiological characteristics. Third, the retrospective nature of the study precludes the establishment of causal relationships.

Another significant limitation is the exclusion of patients who died during follow-up from specific utilization analyses. Given that death is an informative event potentially associated with both PNI and hospitalization outcomes, this approach may have introduced bias. However, to ensure comparable observation time across individuals, admission count analyses were restricted to patients with complete 12-month follow-up. The presence of hospitalization within one year was assessed in the full cohort, including patients who died, partially mitigating this limitation. Nonetheless, future studies employing time-to-event or competing risk models are necessary to more accurately account for informative censoring.

Furthermore, the ROC analysis for 1-year mortality was internally derived and not externally validated. Consequently, the reported AUC and cut-off values may be influenced by optimism bias. Due to sample size constraints, internal validation techniques such as bootstrapping or cross-validation were not performed. Therefore, these findings should be interpreted with caution, and external validation in independent cohorts is essential.

Lastly, nutritional status was evaluated solely through the PNI without comparison to other established nutritional assessment tools.

Future prospective, multicenter studies with larger sample sizes and longitudinal assessments of PNI are needed to elucidate its dynamic prognostic value and to validate our findings.

## Conclusion

This study demonstrated that the PNI is associated with the likelihood of hospitalization and frequency of hospital admissions in patients with cirrhosis. As a straightforward and readily accessible biomarker, the PNI may be beneficial for early risk stratification in clinical practice.

However, given the retrospective design and sample size, additional prospective, multicenter studies are required to confirm these findings and to validate the clinical utility of the PNI.

## Data Availability

The data sets created and/or analyzed during the current study are not publicly available because they contain patient information, but data supporting the findings of this study can be obtained from the corresponding author (AKA) upon reasonable request.
